# Mentalizing Another's Visual World—A Novel Exploration via Motion Aftereffect

**DOI:** 10.3389/fpsyg.2017.01535

**Published:** 2017-09-07

**Authors:** Xuefei Yuan, Nanbo Wang, Haiyan Geng, Shen Zhang

**Affiliations:** ^1^Beijing Key Laboratory of Behavior and Mental Health, School of Psychological and Cognitive Sciences, Peking University Beijing, China; ^2^Department of Psychology, University of Wisconsin-Whitewater Whitewater, WI, United States

**Keywords:** visual perspective-taking, mentalization, aftereffect, motion adaptation, facilitation

## Abstract

Past research on level 2 visual perspective-taking (VPT) has mostly focused on understanding the mental rotation involved when one adopts others' perspective; the mechanisms underlying how the visual world of others is mentally represented remain unclear. In three studies, we addressed this question by adopting a novel VPT task with motion stimuli and exploring the aftereffect on motion discrimination from the self-perspective. Overall the results showed a facilitation aftereffect when participants were instructed to take the avatar's perspective. Meanwhile, participants' self-reported perspective-taking tendencies correlated with the aftereffect for both instructed and spontaneous VPT tasks, when the “to-be-adopted” perspective required the participants to mentally transform their self-body clockwise. Specifically, while facilitation was induced for participants with low self-reported perspective-taking tendencies (e.g., viewing a leftward motion stimulus under another's perspective enhanced subsequent perception of leftward motion from the self-perspective), those with high self-reported perspective-taking tendencies showed an adaptation aftereffect (e.g., viewing a leftward motion stimulus under another's perspective weakened subsequent perception of leftward motion from the self-perspective). For these individuals, the adaptation effect indicated the engagement of direction-selective neurons in processing of the subsequent congruent-direction motion from self's perspective. These findings suggest that motion perception from different perspectives (self vs. another) may share the same direction-selective neural circuitry, and this possibility depends on observers' general perspective-taking tendencies.

## Introduction

Perspective-taking (PT) is the process by which an individual views a situation from another's point-of-view (Galinsky et al., 2008). It is widely adopted in our daily lives to ensure successful social interactions (Tversky and Hard, 2009). For instance, we use perspective-taking to infer how others feel (emotional PT; Ruby and Decety, 2004; Lamm et al., 2007), to represent what others know (cognitive PT; Ruby and Decety, 2003; Apperly et al., 2004) and to make sense of others' actions and intentions (PT of action; Ruby and Decety, 2001; Jackson et al., 2006). One basic and early-developed form of perspective-taking is understanding the visual experience of another agent, known as visual perspective-taking (VPT). The literature has distinguished two levels of VPT: the ability to infer whether an object is visible from another person's line of sight (level 1 VPT; Flavell et al., 1981), and, of particular interest to us, the ability to recognize that a simultaneously visible object can look differently from the different perspectives of the self and another person (level 2 VPT; Michelon and Zacks, 2006).

A considerable amount of research has focused on understanding the nature of level 2 VPT (as opposed to level 1 VPT). These studies usually adopt a paradigm that asks participants to judge the spatial position of an object (e.g., a glove) in disparate scenes (May and Wendt, 2013; Pearson et al., 2013), or report visual content (e.g., the number “6” or “9,” Surtees et al., 2013b) from contradictive perspectives of the self and the avatar. An important feature of level 2 VPT is mentally adopting the spatial position of another person (Surtees et al., 2013a). For example, with increasing angular disparity between the viewpoints of the participants and the avatar, participants' reaction times also increased, suggesting that participants mentally transform themselves to the avatar's position when performing the VPT task (Michelon and Zacks, 2006; Kessler and Thomson, 2010). Although mentally switching into another's spatial point of view is essential, we believe forming a mental representation of the world from that visual perspective is also integral to level 2 VPT.

Nevertheless, most studies have only focused on “adopting another's position.” For example, it has been found that participants' handedness (Gardner and Potts, 2010), motor experience (Steggemann et al., 2011), and the position of the self within the world (Kessler and Thomson, 2010) modulate the difficulty of mental body transformation. Studies were also conducted to understand how level 2 VPT was modulated by participants' gender, socio-cultural background (Mohr et al., 2013; Kessler et al., 2014), emotion, and mental conditions (such as empathy, anxiety and schizotypy; Thakkar and Park, 2010; Gronholm et al., 2012; Todd et al., 2015). These studies as well as studies that explore neural mechanisms of VPT (e.g., David et al., 2006; Mazzarella et al., 2013), however, did not particularly examine the representation of the visual scenes once another's perspective is taken.

How does one visualize objects in that new perspective? Is it the same process as if s/he experiences the stimulus from the self-perspective? Does visual perception from different perspectives (e.g., another's and self's) share some common psychological or neural mechanisms? The present study aimed to answer these questions and understand *the mental representation* of level 2 VPT.

It is difficult to address these questions with past paradigms, for these paradigms either directly asked participants to report the static visual content (the position of an object or number “6 or 9”) under contradictive perspectives of the self and someone else, or indirectly deduced the existence of VPT by demonstrating its interference in visual processing from the self-perspective (Elekes et al., 2016; Surtees et al., 2016). Regardless of approach, participants' reaction time and/or the accuracy of their report in the VPT task were usually the dependent variables. These gross indexes result from the entire processing episode, but do not provide specific information about the mechanism of mental representation under another's perspective in level 2 VPT.

**Figure 1:**
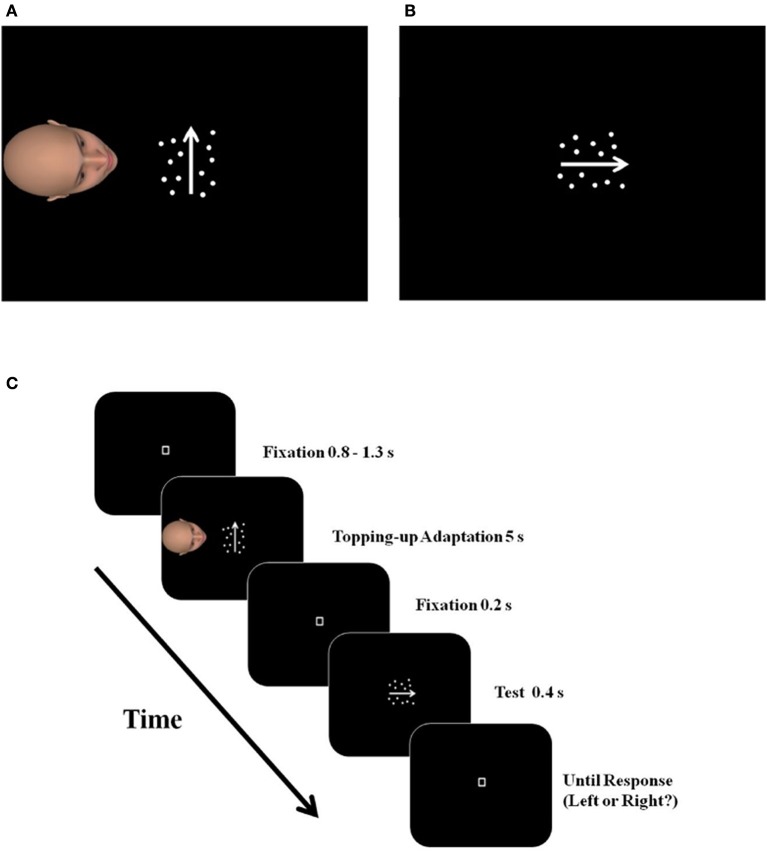
The illustration of a critical trial in the PT condition of Study 1a. After imagining seeing the moving dots from the avatar's perspective for 5 s **(A)**, the subjects made judgments about the dominant moving direction of the test stimulus **(B)**. The arrows in the figure indicate the moving direction of the dots and were not actually presented in the experiments. An example of a complete trial is illustrated in **(C)**.

Instead of static stimuli, we used motion stimuli (i.e., the motion adaptors, see Figure 1A) to examine how VPT affects participants' performance from the perspective of the self in a subsequent motion-direction discrimination task (Figure 1B). This paradigm can answer above research questions, because viewing the motion stimuli from the avatar's perspective for a certain time (5 s in our study) can possibly generate different aftereffects on participants' performance, depending on the mechanisms of mental representation under another's perspective. It should be noted that, the “aftereffect” in this article has a very general meaning, referring to any visual effects from viewing motion stimuli. It does not necessarily mean “the Motion Aftereffect” (usually refers to a motion adaptation effect, Anstis et al., 1998; Huk et al., 2001; Mather et al., 2008).

With this said, the aftereffect could be a motion adaptation effect, an illusion in which after prolonged viewing of motion in one direction, a stationary or ambiguous dynamic test stimulus appears to drift in the opposite direction (Mather et al., 1998; Winawer et al., 2010). It is caused by adaptation of the corresponding neural circuits that reduces subsequent processing of the direction of motion (Wark et al., 2007; Webster, 2011). In our study, this could be the case if motion perceptions from different perspectives (other vs. the self) recruit the same direction-selective neural circuitry, those neurons tuned to leftward movement would become less responsive due to VPT (which is a leftward motion under the avatar's perspective), and thus would weaken one's subsequent processing of leftward motion under the self-perspective. If, however, viewing the motion stimuli under the avatar's perspective does not simulate that in the self-perspective, then such an adaptation aftereffect is unlikely to occur.

Thus in Study 1 we explicitly instructed participants to take the avatar's perspective to view leftward/rightward motion adaptors, and measured their performance, i.e., the possible aftereffect, in a subsequent leftward/rightward motion-direction discrimination task under the self-perspective. We also conducted Study 2 to try to replicate the main findings of Study 1.

We believe that the aftereffect ties to participants' PT abilities. It has been found that when participants were asked to judge the relative direction of a static object (Thakkar and Park, 2010), their' self-reported PT tendencies positively correlated with the efficiency in completing that VPT task. Such self-reported PT tendency should predict behavior in VPT task with motion stimuli too, since it indicates participants' general ability of adopting another's perspective, independent of visual stimuli (Davis, 1980). Meanwhile, individual difference in visual motion perception has been found in recent literature: following the presentation of the same stimulus, some participants demonstrated motion facilitation, while others demonstrated motion adaptation (Takeuchi et al., 2017). Thus, we investigated whether the aftereffect following VPT with motion stimuli would vary along with participants' self-reported PT tendencies. Due to their stronger ability of perspective-taking, we assume people with high self-reported PT tendencies would be immersive when mentalizing other's visual world, therefore we predict these people exhibit an adaptation effect on subsequent motion perception. For people with low self-reported PT tendencies, their aftereffect might be weaker or a different kind.

To measure participants' PT tendencies, we adopted the PT subscale of the Interpersonal Reactivity Index (IRI scale; Davis, 1980). The IRI scale consists of four seven-item subscales, and each measures an aspect of the global concept “empathy.” The PT subscale fits the aim of our research well, for it contains items assessing people's spontaneous tendencies to take other people's perspectives and see things from their points of view in everyday life (e.g., “I sometimes try to understand my friends better by imagining how things look from their perspective” and “I try to look at everybody's side of a disagreement before I make a decision”).

Recent literature showed that level 2 VPT can be spontaneously induced (Elekes et al., 2016; Surtees et al., 2016). For example, when participants had to report the visual content of a number shown on the table from their own view while another person was sitting across the table from them, their reaction time was longer when the number was “6” or “9” rather than “0” or “8,” which was thought to result from the interference of participants' spontaneous VPT on their own perspective processing (Elekes et al., 2016; Surtees et al., 2016).

Unlike Study 1 and 2 that examine instructed perspective-taking by deliberately requiring participants to take another person's viewpoint, we conducted Study 3 to explore spontaneous perspective-taking and its possible aftereffect. Since literature comparing explicit vs. implicit processing usually demonstrated a weaker effect for the latter (Jiang and He, 2006; Jiang et al., 2009), we expect a similar but weaker effect from spontaneous VPT with motion stimuli.

In summary, with a novel motion-adaptation paradigm, we conducted three studies to investigate the mechanisms of mental representation under others' perspective. We aim to examine whether the direction-selective neural circuits engaged in self-perspective processing are also involved in level 2 VPT, and whether the approaches people employ to adopt another's perspective are correlated with their self-reported PT tendencies.

## Study 1 (a and b)

### Study 1a

Study 1a was conducted as the main experiment to examine the mental representation of instructed level 2 VPT.

Participants were asked to take the avatar's perspective to view a motion stimulus comprised of a set of light-colored dots on a dark background, moving leftward or rightward from the avatar's perspective (but upward or downward from the participant's perspective), and subsequently complete a leftward/rightward motion-direction discrimination task from the self-perspective (Figure 1B).

After mentally transforming themselves to the avatar's position, if participants use the same populations of neurons to process the moving dots during VPT as they do in their own perspective, prolonged viewing of a motion stimulus (e.g., moving upward) from the avatar's perspective (moving leftward, Figure 1A) will lead to an adaptation aftereffect for motion in that direction (leftward). As a result, participants' discrimination of the subsequent motion stimulus in the adapted direction will weaken, manifested as a higher probability of participants' reporting the opposite direction (rightward) in the subsequent discrimination task from the self-perspective.

We also explored whether the occurrence of the aftereffect relates to people's PT tendencies, in that higher PT tendencies predicts a stronger adaptation effect.

#### Method

##### Participants

Seventeen students with normal or corrected-to-normal vision were recruited from Peking University for this study and received monetary compensation or course credits for their participation. Data from one participant were excluded because of extremely low accuracy in his performance (beyond three standard deviations from the group mean). Results from the remaining 16 participants (6 females and 10 males; *M*_*age*_ = 22.7 years, *SD* = 2.6) were included in the final analyses. All studies reported in this paper were approved by the Ethics Review Committee of Peking University.

##### Materials

*The computer task*. The program used for the computer task was generated by Matlab2011 with the Psychtoolbox 3. All stimuli, described below, were presented on a 19-in Viewsonic Professional Series P97f+ (1,024 × 768 at 75 Hz) monitor connected to a computer running Windows XP.

The fixation point was an outline of a light-gray square presented at the center of the screen, subtending 0.4° × 0.4° of visual angle, with a luminance of 6.65 cd/m^2^.

Each motion stimulus consisted of three sequences of randomly distributed Gaussian, anti-aliased white dots with interleaving frames (60 cd/m^2^ at maximum contrast; 0.06 deg at half-height, with a 5 dots/deg^2^ density; Shadlen and Newsome, 2001; Roitman and Shadlen, 2002). These dots drifted at a speed of 8 deg/s within a region subtending 4° × 4° of visual angle against a black background at the center of the computer screen (0.05 cd/m^2^). These dots moved vertically as a motion adaptor, but moved horizontally as a test stimulus (see Procedure). When they were a test stimulus, not all but only a percentage of dots were moving coherently (“motion coherence,” Newsome and Pare, 1988). A total of nine coherence levels were used, randomly varied from trial to trial: 0, ±5, ±10, ±20, and ±40%, where the negative and positive signs indicate the leftward and rightward motion, respectively.

The avatar was an average Eastern Asian face with neutral emotional expression and indifferent gender characteristics, generated by FaceGen 3.4.1 (Copyright 2009, Singular Inversions Inc.). Facing the motion stimulus, the avatar was located 6° horizontally away from the center of the screen to the left, subtending 7° × 7°of visual angle. The participants had a top view of the avatar's head (Figure 1).

*The pt measure*. Participants' PT tendencies was measured by the Perspective-Taking (PT) subscale of IRI (Davis, 1980).

##### Procedure

Participants individually completed the computer task with their heads supported by a chin rest, at a viewing distance of 57 cm from the computer screen. They also completed the PT measure at the end of the experiment.

There were three experimental conditions in the computer task: Perspective-Taking (PT), Adaptor Only (AO), and baseline. There was a practice block of trials within each condition. Both the Perspective-taking (PT) and the Adaptor Only (AO) conditions included 10 blocks of trials with a motion adaptor moving upward or downward (5 blocks for each direction), followed by a presentation of test stimulus in each trial. The baseline condition, however, only had 5 blocks of trials with only the presentation of test stimulus included in each trial. Each block had 45 critical trials across all three conditions, and additional 10 catch trials in the PT condition as well as 5 catch trials in the AO condition (see below the description of the conditions for detail). No catch trials was presented in the baseline condition.

Participants went through a total of 25 blocks of trials in 3 days, to reduce fatigue from working on the computer task each day. Two PT blocks and two AO blocks (with either upward or downward adaptor in each block), as well as one baseline block, were run on the first day. The number of blocks was doubled for each condition on the following 2 days, with all blocks run in a random order on each day.

*Pt condition*. At the beginning of each block, there was a 20 s presentation of the motion adaptor (pre-adaption, 100% motion coherence) and 3 warm-up trials (not included in the final analysis) to familiarize participants with the procedure. Then the participant went through 45 critical trials (nine levels of motion coherence; five trials at each level).

A critical trial (Figure 1C) started with a fixation of random duration (0.8–1.3 s), and was followed by a 5 s-presentation of a motion adaptor (topping-up adaption, 100% motion coherence), moving upward or downward from the self-perspective. At the same time, the avatar's head was presented on the left side of the adaptor (Figure 1A). Participants were instructed to continuously imagine themselves looking at the motion adaptor from the avatar's perspective. After a 0.2 s fixation-only interval, a horizontal-moving (from the self-perspective) test stimulus was presented for 0.4 s, at one of the nine coherence levels. Participants were asked to make a two-alternative forced-choice (2-AFC) judgment of the dominant direction of the test stimulus (either left or right) as accurately as possible (Figure 1B). Their responses prompt the beginning of the next trial.

Each block also included two types of “catch” trials, randomly mixed with the critical trials. Specifically, five “motion acceleration” trials were used to ensure participants were attending to the adaptor. In such a trial, the speed of the motion adaptor increased abruptly from 8°/s to 16°/s, a change that required the participants' immediate response by pressing the “N” key on the keyboard. Five “closed eyes” trials were used to ensure the participants were paying their attention to the avatar. In such a trial, the eyes of the avatar closed at a random time point between 1 and 3 s after the appearance of the avatar. Participants were asked to press the “V” key as soon as they detected this change. A failure to respond within 1 s prompted a warning message on the center of the screen for 0.6 s and the termination of the current trial. No test stimuli was presented in either “motion acceleration” or “closed eyes” trials. All participants in study 1a as well as in the rest of the studies reported in this paper completed the catch trials with above 90% accuracy, suggesting that they paid sufficient attention to the motion adaptor and the avatar's face.

*Ao condition*. The AO condition was the same as the PT condition, except that neither avatar nor “closed eyes” catch trials were included.

*Baseline condition*. The baseline condition did not have the avatar nor any adaptor, but only presented the test stimulus 0.2 s after the fixation for participants to judge it's direction, as a measure of the participants' baseline motion discrimination sensitivity.

Participants completed the PT measure at the end of the experiment.

##### Data analysis

**Figure 2:**
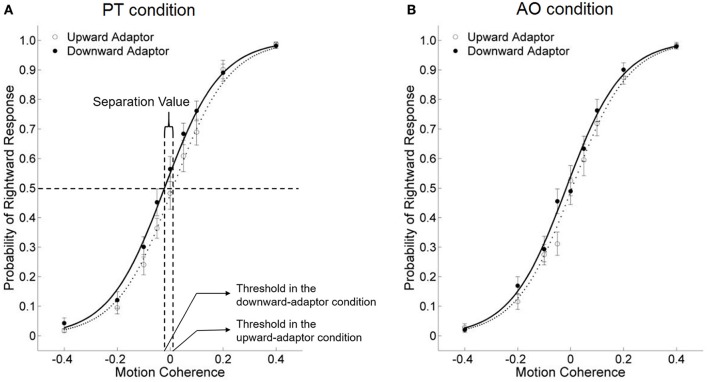
The effects of viewing motion adaptors on perceived direction of test stimuli under the PT condition **(A)** and the AO condition **(B)** in Study 1a, for all participants combined. The threshold of the test stimuli was quantified as the amount of motion coherence that yielded 50% “rightward” responses on the psychometric function curve. Meanwhile, the separation value was quantified as the difference between the two thresholds in the upward and downward adaptor conditions. If the separation value is positive (i.e., the upward-adaptor curve was on the left side of the downward-adaptor curve), it indicates an adaptation effect; if the separation value is negative, it indicates a facilitation effect. The abscissa refers to the motion coherence, with positive values for rightward motion and negative values for leftward motion. Error bars are ±1 SEM.

Participants' probabilities of “rightward” responses following upward adaptors vs. downward adaptors were estimated by logistic regression analysis (see Appendix for model fits). The dependent variable of our study was the threshold of the test stimuli, which was the amount of motion coherence that yielded 50% “rightward” responses on the psychometric function curve (see a graphic illustration in Figure 2A), and was tested against a repeated measure ANOVA.

Meanwhile, the horizontal separation value, which was the difference between the two thresholds in the upward and downward adaptor conditions, was used as the index of the aftereffect from perceiving motion stimuli under the avatar's perspective, and correlated with participants' self-reported PT scores.

If the aftereffect did not exist, the two curves would overlap. If the separation value is positive (the threshold of the downward-adaptor curve is larger than that of the upward-adaptor curve), it indicates an adaptation effect: for instance, a smaller probability of “rightward” response after viewing a downward, rather than an upward adaptor. In contrast, a negative separation value (a smaller threshold of the downward-adaptor curve than the upward-adaptor curve) indicates a facilitation effect, which means viewing the motion adaptor facilitated subsequent motion perception in congruent-direction, for instance, a larger probability of “rightward” response after viewing a downward, rather than an upward adaptor.

#### Results

To examine whether response bias existed among participants, we conducted a one-sample *t*-test on the motion coherence of the test stimuli that yielded 50% “rightward” responses against 0. The result was not significant, *t*_(15)_ = −0.349, *p* = 0.732, indicating participants' unbiased responses for discriminating the motion direction of the test stimuli. Although only reported here, the same test was conducted for all studies in this paper and none of the results was significant (Table 1).

**Table 1:** Means (SE) of the motion coherence of the test stimuli that yielded 50% “rightward” responses in the baseline condition of each study.

**Study 1a**	**Study 1b**	**Study 2**	**Study 3a**	**Study 3b**
−0.006 (0.016)^*^	−0.019 (0.011)	0.004 (0.017)	−0.017 (0.011)	−0.015 (0.010)

* *Each mean value was tested against zero with a two-tailed, one-sample t-test. All ps > 0.1*.

Taking a general overview of the data from the experimental conditions, we found two categories of aftereffects. Some participants showed a positive separation value between the two psychometric function curves, indicating an adaptation effect, whereas some others showed a negative separation value, which indicates a facilitation effect.

**Figure 3:**
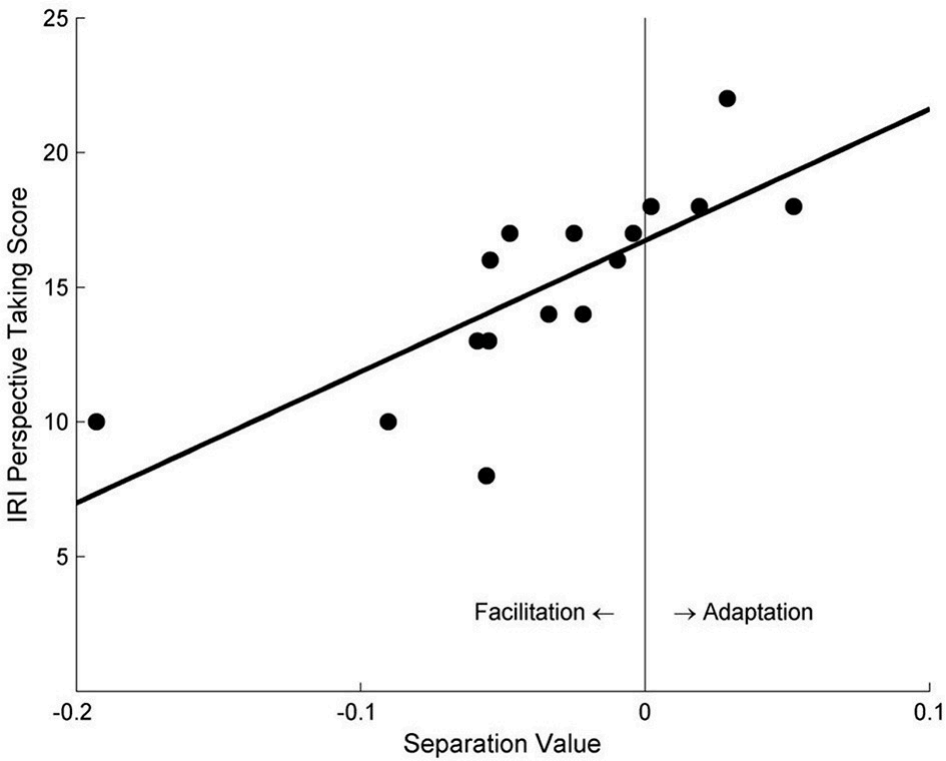
The correlation between the separation values of psychometric function curves and participants' self-reported PT scores in Study 1a. Negative values of abscissa indicate facilitation effects, whereas positive values indicate adaptation effects.

Participants' separation value between the two psychometric function curves was found to significantly correlate with their self-reported PT tendency scores, for both the PT condition (*r* = 0.76, *p* < 0.001) and the AO condition (*r* = 0.53, *p* = 0.033). Specifically, participants with lower PT scores demonstrated a facilitation effect, whereas participants with higher PT scores showed an adaptation effect (Figure 3).

Across all participants, we conducted a 2 × 2 repeated measure ANOVA on the thresholds of the test stimuli, with Condition (PT vs. AO) and Motion Adaptor (upward vs. downward) as within-participant factors. Neither the main effect of Condition nor the interaction was significant, [*Fs*_(1, 15)_ < 1, η^2^ = 0.001 and η^2^ = 0.060]. The only significant main effect is Motion Adaptor, *F*_(1, 15)_ = 6.998, *p* = 0.018, η^2^ = 0.318. These results indicates a facilitation effect for both the PT and the AO conditions (Figures 2A,B), but the two facilitation effects do not differ from each other.

### Study 1b

We speculated that the facilitation effect found in the AO condition in Study 1a was a carryover effect. Since blocks of different conditions were interleaved and the avatar always appeared on the left side of the visual scene, participants' perspective-taking tendencies induced by the PT blocks might transfer to the AO blocks that did not have the avatar. Literature also showed that intensive practice of mental rotation activates memory mechanisms, and possibly leads to the extraction of the rotated representation of the stimuli directly from memory without actual mental rotation (Tarr and Pinker, 1989).

Alternatively, one could argue that, instead of perspective-taking, the upward/downward adaptors *per se* affected the discrimination sensitivity for the horizontal motion stimuli for both the PT and the AO blocks. To test and rule out this explanation, we conducted Study 1b that only included the AO condition and the baseline condition. Without the PT condition, we expected no aftereffect and therefore no separation of the psychometric curves between the upward and downward adaptors in the AO condition.

#### Method

##### Participants

Fourteen students (7 females and 7 males; *M*_*age*_ = 20.6 years, *SD* = 1.8) from Peking University with normal or corrected-to-normal vision participated in Study 1b for payment or credits.

##### Materials and procedure

Materials and procedures were the same as in Study 1a, except that no PT condition was used. The participants completed 10 AO blocks (4 on the first day and 6 on the second day) with either upward or downward adaptor in each block. They also completed 5 baseline blocks (2 on the first day and 3 on the second day). The AO and baseline blocks on each day were mixed and run in a random order.

#### Results

Psychometric curves were fitted using the same method as in Study 1a. No significant separation was found between the motion sensitivity curves with the upward vs. downward adaptors in the AO condition (Mean = 0.012, 95% CI = [−0.015, 0.039]), *t*_(13)_ = 0.96, *p* = 0.35, which suggests no aftereffect was found.

This result demonstrates that upward/downward motion stimuli *per se* have no selective impact on participants' processing of subsequent leftward/rightward motion stimuli, which is consistent with previous studies (Verstraten et al., 1994; Grunewald and Lankheet, 1996). Therefore, the facilitation effect observed in the AO condition in Study 1a was likely transferred from the PT blocks that participants also went through.

### Discussion

How people represent objects or visual scenes after taking another's perspective has rarely been studied before. We used a new paradigm to address this issue by examining the aftereffect of mentalizing another's visual world on participants' subsequent visual motion perception.

The results clearly demonstrated the existence of VPT, showing that participants' VPT had an impact on their subsequent performance in a motion discrimination task, manifested as the separation value. Moreover, the separation value was significantly correlated with participants' self-reported perspective-taking tendencies, gradually increasing from negative (i.e., a facilitation effect) to positive (i.e., an adaptation effect) as the perspective-taking tendency increases.

Although we had anticipated a main effect of Condition and an interaction effect, neither effect was found except a main effect of Motion Adaptor. This means that the aftereffects found in PT and AO conditions were the same. These aftereffects must result from performing the VPT task, for in Study 1b when there was no avatar and thus no VPT, watching vertical motion stimuli alone did not induce any aftereffect in the subsequent motion discrimination task.

Participants' varied aftereffects may reflect different abilities and/or processing mechanisms in performing the VPT task. Note our VPT task is quite different from previous research. For example, participants had to perform complex mental movement in the 3-D space to take the avatar's viewpoint, compared with the common setups that only require a 2-D mental transformation. Moreover, perceiving motion stimuli from another's perspective and sustaining that mental representation was more complicated than perceiving a static object. Therefore, not all people were able to complete this task to the same degree.

The appearance of an adaptation effect suggests that those participants were not only adopting the avatar's perspective, but also constantly processing the motion adaptor from that perspective, and perceived a leftward/rightward motion during the mental representation. At the cortical level, this effect means that the motion perception from another's perspective recruited the same direction-selective sensory neurons as did the motion perception from the self-perspective. These neurons were tuned to the VPT stimuli, and thus less responsive to the subsequent same-oriented motion perceived from the self-perspective. Participants experiencing a facilitation effect, however, likely did not perceive the motion adaptors as moving left or right, probably because they failed to adopt the avatar's position, or vividly mentalize the avatar's visual world. These participants might have used different mechanisms instead.

Only the participants with higher perspective-taking tendencies tended to demonstrate an adaptation effect; people with lower PT scores were more likely to experience a facilitation effect (Figure 3). This may reflect participants' differing abilities to simulate other people's motion perception when taking their visual perspectives. People with high PT tendencies may perform perspective-taking frequently in their daily lives, and become skillful perspective-takers with rich experiences. They may be able to efficiently use cognitive resources to deal with high-demand tasks like PT, in that it might be easier for these people to be “immersive” during VPT: they tend to take others' perspective as if they actually stand in the other's place, and retain that perspective, thus producing the motion adaptation effect later on.

Conversely, people with low PT tendencies are likely to have fewer real life perspective-taking experiences, and therefore are not as skillful in preforming PT. Consequently, they may not be able to or have adequate cognitive resources to consistently imagine themselves being in the avatar's perspective during the prolonged VPT period, or to suppress their own viewpoint when processing the motion adaptors. Instead, they may be more likely to adopt different strategies that are less cognitively resource-demanding, such as setting up prior rules to process visual stimuli under VPT (for example, “If I were in the avatar's position, then the motion stimuli should be moving leftward/rightward in that view,” Michelon and Zacks, 2006). Processing of this inference would be more economical than creating a concrete visual image to represent another's visual world, but it would also prime the participant and facilitate processing of the subsequent congruent-direction motion (leftward/rightward) in their own perspective (Wohlschläger, 2000; Kanai and Verstraten, 2005), therefore inducing a facilitation effect.

Thus, the overall facilitation effect found in our study can be accounted for by the general use of such a quick rule of “leftward” or “rightward,” which then biased the participants' responses to the corresponding direction of motion in the subsequent motion-discrimination task.

## Study 2

Previous studies found that the processing of social cues has a right-hemisphere bias. People are faster and more accurate in processing social cues located in their left rather than right visual field (Greene and Zaidel, 2011; Semrud-Clikeman et al., 2011). Meanwhile, visual face processing also occurs differently in left and right hemispheres, in that the left hemisphere is involved in processing “low-level” face semblance, whereas the right hemisphere is involved in performing categorical “deep” analyses such as face/non-face information (Meng et al., 2012).

Unlike in Study 1a where the avatar always appeared in the left visual field, in Study 2 we manipulated the location of the avatar to be either in the left or right visual field. Meanwhile, we divided participants into high and low PT groups according to a median split of their PT subscale scores. We explored whether our findings in Study 1 could be replicated in Study 2, and whether the aftereffect still exists when the avatar is in the right visual field.

Since motion adaptors alone did not impact participants' performance in the AO condition in Study 1b, we only included the baseline condition and the PT condition in Study 2.

### Method

#### Participants

Twenty-three students from Peking University with normal or corrected-to-normal vision participated in Study 2 for payment or credits. Data from one participant were excluded because of extremely low accuracy in his performance (beyond three standard deviations from the group mean). The results from the remaining 22 participants (9 males, 13 females; *M*_*age*_ = 22.2 years, *SD* = 2.1) were included in the final analyses.

#### Procedure

Participants completed 8 blocks of computer trials each day for 2 days. On each day, the first and eighth blocks were the baseline condition, without the avatar nor a motion adaptor. The other six blocks were the PT condition, in which the position of the avatar remained constant for the day (either left or right relative to the center of the computer screen), and then switched to the opposite side on the other day. Meanwhile, the motion direction of the adaptor in the first three blocks, either upward or downward, was opposite to that in the second three blocks. The position of the avatar on the first day and the direction of the adaptor in the first three PT blocks on each day were balanced across participants. The same critical trials and catch trials (except for varied avatar position between PT blocks) were included in each block as in Study 1a.

#### Data analysis

Data analysis was conducted the same way as in Study 1a. In addition, since this study had more participants, they were classified as high and low PT tendency groups based on a median split of their self-reported PT scores (Mdn = 16).

### Results

**Figure 4:**
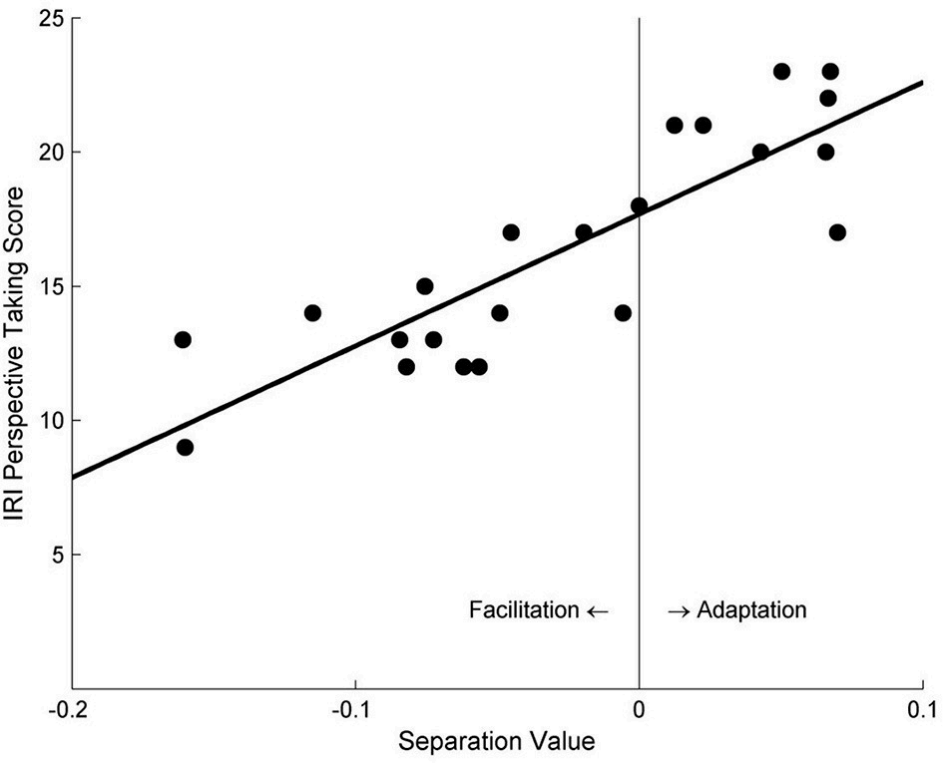
The relationship between the separation values of motion sensitivity curves and the self-reported PT scores under the PT + Left Avatar condition in Study 2.

Consistent with Study 1a, participants' self-reported PT tendency was found to strongly correlate with the separation value of the psychometric curves in the PT + Left Avatar condition, *r* = 0.86, *p* < 0.001 (Figure 4). However, no significant correlation was found in the PT + Right Avatar condition, *r* = 0.13, *p* = 0.55.

We performed a 2 (Avatar Position: left vs. right) × 2 (PT Group: high vs. low) × 2 (Motion Adaptor: upward vs. downward) repeated measure ANOVA on the threshold of the test stimuli. The main effect of Motion Adaptor was marginal significant, with lower threshold for the downward adaptors, *F*_(1, 10)_ = 4.235, *p* = 0.067, η^2^ = 0.298, indicating a facilitation effect when judging the direction of test stimuli, as in Study 1a. The interaction effect of PT Group and Motion Adaptor was significant, *F*_(1, 10)_ = 11.691, *p* = 0.007, η^2^ = 0.539. More importantly, the three-way interaction between Avatar Position, PT Group and Motion Adaptor was also marginally significant, *F*_(1, 10)_ = 3.726, *p* = 0.082, η^2^ = 0.271.

**Figure 5:**
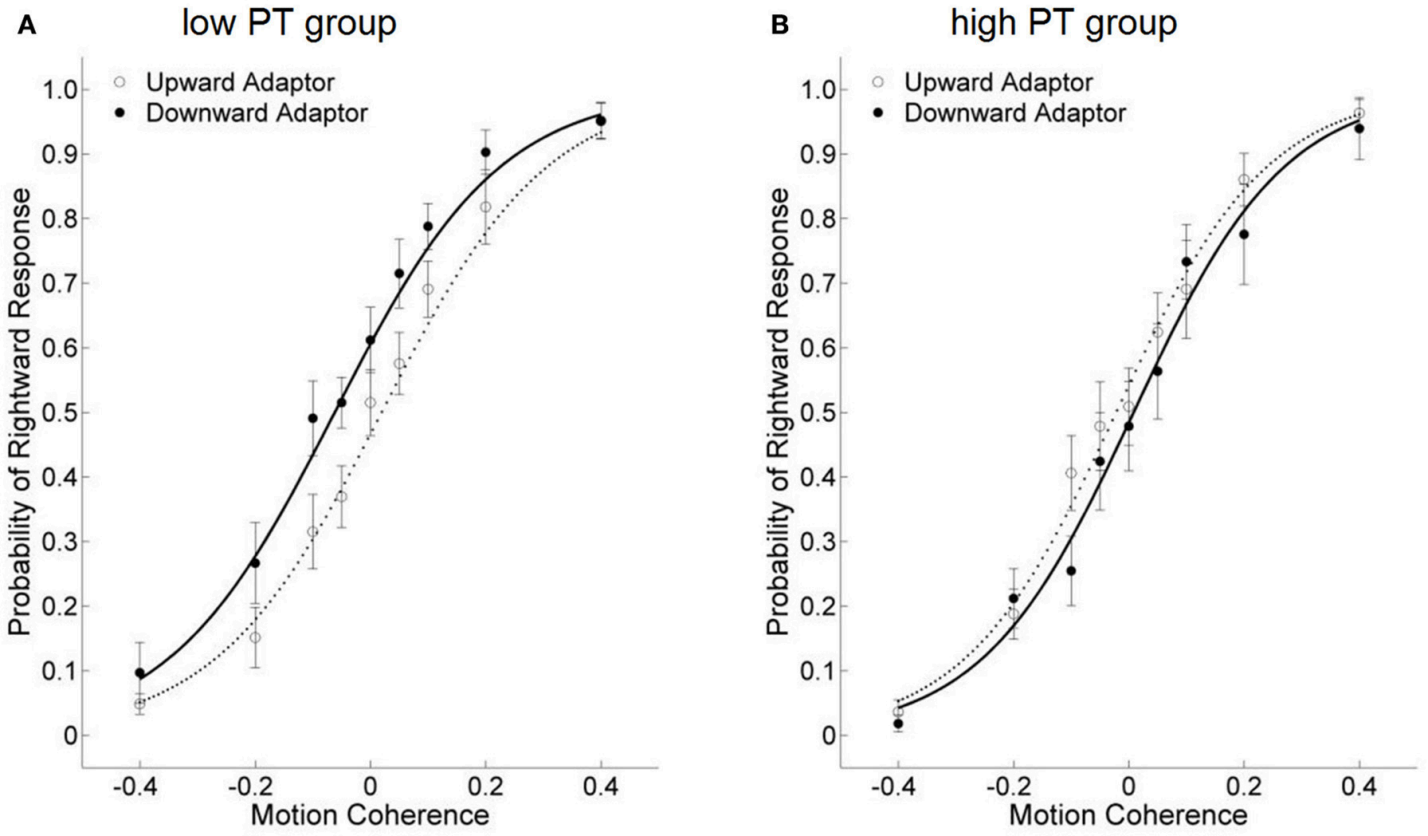
The effects of viewing motion adaptors on perceived direction of test stimuli under the PT + Left Avatar condition in Study 2. **(A,B)** show data from the low and high self-reported PT tendency groups, respectively.

Pairwise comparisons were conducted to understand this three-way interaction. Specifically, in the PT + Left Avatar condition, participants with low self-reported PT scores demonstrated a facilitation effect in the subsequent motion processing (Figure 5A), indicated by a significant lower threshold in the downward-adaptor condition than the upward-adaptor condition, Mean difference = −0.084, 95% CI = [−0.115, −0.053], *p* < 0.001. However, participants with high self-reported PT scores showed a motion adaptation effect (Figure 5B), indicated by a significant higher threshold in the downward-adaptor condition than for upward-adaptor condition, Mean difference = 0.031, 95% CI = [0.004, 0.058], *p* = 0.027. No significant result was found in the PT + Right Avatar condition.

#### Cross-study analysis

Because the procedure of Study 1a (the PT condition) and Study 2 (the PT + Left Avatar condition) was exactly the same, to further validate our results with a larger sample size, we did the same median split with participants in Study 1a and then combined the data from these two conditions. The same correlation analysis between the separation value and participants' PT scores revealed consistent result: among a total of 38 participants, Pearson *r* = 0.819, *p* < 0.001.

The combined data was also subjected to a 2 (PT Group: high vs. low) × 2 (Motion Adaptor: upward vs. downward) repeated measure ANOVA on the threshold of the test stimuli, and revealed a significant effect of Motion Adaptor [*F*_(1, 18)_ = 19.202, *p* < 0.001, η^2^ = 0.516]. This effect was further quantified by the significant interaction [*F*_(1, 18)_ = 43.310, *p* < 0.001, η^2^ = 0.706]. Pairwise comparisons showed a significant adaptation effect for participants in the high PT group; but a significant facilitation effect was found for those in the low PT group. These findings were demonstrated by a significant higher threshold for the downward-adaptor condition in the high PT group (Mean difference = 0.019, 95% CI = [0.000, 0.037], *p* = 0.046) but a significant lower threshold for the downward-adaptor condition in the low PT group (Mean difference = −0.078, 95% CI = [−0.102, −0.055], *p* < 0.001).

### Discussion

Consistent with the correlational results in Study 1a, we found an adaptation effect on the subsequent motion probes among participants with higher self-reported PT tendencies, and a facilitating effect among those with lower self-reported PT tendencies, which again indicates different mentalizing abilities or strategies between different participants. Importantly, these findings were replicated in the cross-study analysis of Study 1a and 2, with more participants. However, these significant aftereffects of the adaptors occurred only when the avatar was in the left visual field, when participants performed mental body transformation in the process of adopting the avatar's position.

Two possible reasons might account for this result. On the one hand, these findings may indicate that, as the cue for mental body transformation, the avatar was rendered effective only in the left visual field, similar to previous findings about right-hemisphere lateralization of processing social cues (Greene and Zaidel, 2011; Semrud-Clikeman et al., 2011). For example, although averted gaze can trigger automatic gaze following (Friesen and Kingstone, 1998; Friesen et al., 2004; Xu et al., 2011), only stimuli presented in the left visual field induced such effects (Greene and Zaidel, 2011).

On the other hand, given that participants' perspective transformation necessarily involves clockwise or counterclockwise mental-body rotation to take the avatar's position, the effect found in the left visual field may also be due to the advantage of clockwise mental rotation. Clockwise mental rotation is related to faster reaction times and higher performance accuracy than counterclockwise mental rotation (Liesefeld and Zimmer, 2011). This indicates that performing counterclockwise mental-body transformation is more difficult and resource demanding.

Future research should explore exactly which reason is responsible for the null result of VPT on the subsequent motion discrimination when the avatar was in the right viusal field.

## Study 3 (a and b)

### Study 3a

In real life, perspective-taking often occurs spontaneously. In Study 3a we examined whether mental representation of another's visual world, as reflected by the aftereffects found in Study 1 and 2, can also be observed when participants were not deliberately instructed to take the avatar's perspective.

#### Method

##### Participants

Twenty-two students (10 males, 12 females; *M*_*age*_ = 20.2 years, *SD* = 1.6) from Peking University with normal or corrected-to-normal vision participated in Study 3 for payment or credits.

##### Procedure

Materials and procedures were the same as Study 2, with the exception that participants were not instructed to take the avatar's perspective in the PT condition. Instead, they were just told to judge the direction of the test stimuli as in the baseline condition. The same two types of catch trials were also contained in the PT condition as in Study 2.

#### Results

**Figure 6:**
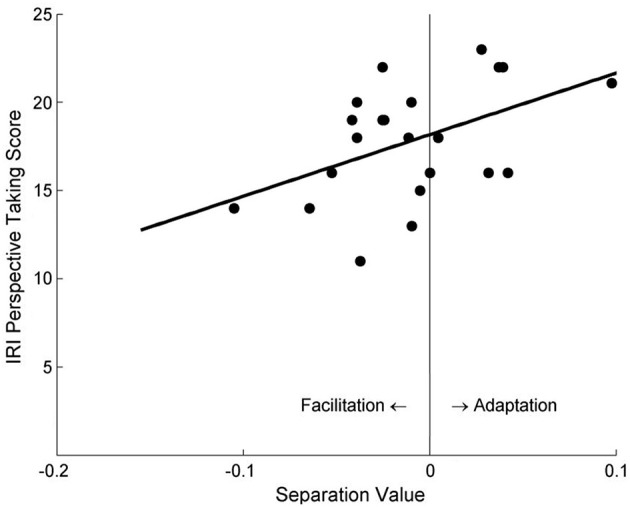
The relationship between the separation values of motion sensitivity curves and self-reported PT scores under the PT + Left Avatar condition in Study 3a.

Data analysis was conducted in the same way as in Study 2. The only significant finding regarding the aftereffect was its significant correlation with participants' self-reported PT scores in the PT + Left Avatar condition, *r* = 0.48, *p* = 0.023 (Figure 6), but not in the PT + Right Avatar condition, *r* = −0.34, *p* = 0.128.

### Study 3b

When we investigated spontaneous VPT in Study 3a, since there was no explicit instructions on adopting the avatar's perspective nor manipulations to ensure participants to do so, one might argue that the significant correlation result had other explanations. To further test whether this finding was indeed due to spontaneous VPT, we conducted Study 3b following the exact procedure as in Study 3a, except that the eyes of the avatar were closed.

Gaze is a powerful modulator of spontaneous VPT. Knowing someone is gazing at something, or knowing they are not gazing at something, can increase or decrease the probability of spontaneously taking that person's visual perspective, respectively (Teufel et al., 2010; Furlanetto et al., 2013, 2015). Therefore, if participants indeed spontaneously take the perspective of the avatar, then when the avatar closes its eyes, any effect of perspective-taking should diminish. Study 3b aimed to test this speculation.

#### Methods

##### Participants

Twenty-two students from Peking University with normal or corrected-to-normal vision participated in Study 3b for payment or credits. Data from one participant were excluded because of extremely low accuracy in his performance (beyond three standard deviations from the group mean). The results from the remaining 21 participants (10 males, 11 females; *M*_*age*_ = 22.2 years, *SD* = 2.1) were included in the final analyses.

##### Procedure

Materials and procedures were the same as in Study 3a, except that the avatar's eyes were closed in critical trials but opened in catch trials. In such catch trials, participants had to press the “V” key as soon as they detected the opening (instead of closing) of the avatar's eyes. The “motion acceleration” catch trials remained the same as in Study 3a.

#### Results

Data analysis followed the convention in Study 3a. We did not find any significant aftereffect, nor significant correlation between aftereffect and self-reported PT scores, in either the PT + Left Avatar or PT + Right Avatar conditions (Left: *r* = −0.22, *p* = 0.35; Right: *r* = 0.20, *p* = 0.38).

#### Discussion

In Study 3a, participants were not instructed to take the avatar's perspective. However, the correlation between the separation value and participants' self-reported PT scores was (still) significant, and its direction was similar to that in the instructed VPT. This result suggests that even in the absence of specific perspective-taking prompts, the existence of the avatar automatically triggered VPT. People with higher PT tendencies may be especially more proactive in taking other's perspective compared to people with lower PT tendencies. Further, when avatar's eyes were closed in Study 3b (and thus participants understood that the avatar could not view the stimuli), spontaneous VPT diminished, shown by a lack of aftereffect and absence of a correlation between the aftereffect and self-reported PT scores. These results provide further evidence that the correlation in Study 3a was due to spontaneous VPT.

However, we did not find a significant adaptation or facilitation effect when dividing the participants based on the median split of their PT scores. Study 2 found a larger effect compared to Study 3a [Fisher Z transformation showed that *Z*_Study2_ = 1.293 and *Z*_Study3a_ = 0.523; a comparison between the two independent correlation coefficient results in *z* (42) = 2.373, *p* = 0.009]. These findings may indicate a weaker effect from spontaneous VPT than intentional VPT, consistent with the results of pervious research which compared implicit processing to explicit processing (Jiang and He, 2006; Jiang et al., 2009).

## General discussion

When performing a level 2 VPT task, people mentally adopt another's spatial position, and perceive the visual scene from that perspective (Michelon and Zacks, 2006; Kessler and Rutherford, 2010). Previous research aimed to understand the mental transformation involved when people mentally take another's position, but rarely examined the characteristics of the mental representation if one is already under another's perspective. By examining how visual processing during VPT affected participants' performance in a subsequent motion discrimination task, our study, for the first time, revealed that individuals with varying levels of self-reported PT tendency take different approaches when representing another's visual world.

Overall, we found a significant facilitation effect when participants were instructed to take other's perspective in Study 1. Moreover, across all three studies exploring both instructed and spontaneous VPT, the aftereffects of VPT were consistently correlated with people's self-reported daily PT tendencies. In line with our hypothesis, in this VPT task with motion stimuli, people with higher self-reported PT tendencies tended to show an adaptation effect (i.e., processing leftward motion from the avatar's perspective *weakened* subsequent processing of leftward motion under the self-perspective), whereas those with lower self-reported PT tendencies showed a facilitation effect (i.e., processing leftward motion under the avatar's perspective *facilitated* subsequent processing of leftward motion under the self-perspective), and this correlation existed when people both intentionally and spontaneously took another's perspective.

The mechanisms accounting for adaptation and facilitation are completely different: motion adaptation is caused by the reduced response of specific direction-selective neurons, whereas facilitation is caused by a priming effect, where perceiving former stimulus eases processing of subsequent similar stimuli. Such varied aftereffects have been reported in a recent study, but only upon perceiving an ambiguous motion stimulus after a brief exposure to a moving adaptor from the self-perspective (Takeuchi et al., 2017). Our study with prolonged viewing of motion adaptation stimuli, however, revealed different types of processing in level 2 VPT.

One explanation lies in different mentalizing abilities of people with high and low PT tendencies. People with high PT tendencies may have better mentalizing abilities than their low-PT-tendency counterparts, because of different daily experiences of PT, i.e., they are more used to taking another's perspective in their social life, and their PT process may be more “automatic” and less resource demanding. Another related interpretation is that the processing strategies may be different. Participants with high PT tendencies may indeed put themselves in the avatar's perspective, whereas participants with lower PT tendencies may have used an alternative strategy such as forming a verbal inference about what the stimulus should look like from the avatar's perspective.

As a result, those high-PT-tendency people might be more likely to form a detailed visual representation (which is highly demanding of cognitive resources) just as they see the stimulus from their own perspective, and this representation taxed and weakened the activities of direction-selective neurons, causing a motion adaptation aftereffect. Low-PT-tendency people, however, might either lack the ability or are not efficient in using cognitive resources to perform mental representation, so they used inferential strategies instead, inducing a facilitation effect. Meanwhile, the different processing strategies may not be mutually exclusive, as indicated by the gradually changing separation value of the two curves with different adaptors as well as the positive correlation between separation value and individual PT scores. The PT tendency possibly predicts the different extent to which different processing strategies were adopted.

Note if not for the paradigm we adopted, we won't be able to reveal these differences. Instead of collecting participants' immediate responses upon taking the avatar's perspective, our novel paradigm requires the participants to hold that perspective, and thus allows a prolonged mental representation phase to generate aftereffects in subsequent motion discrimination. Meanwhile, in our paradigm the direction of motion adaptors was orthogonal when perceived from the participants' perspective and the avatar's perspective (i.e., the avatar was on the left or the right side of the screen and the motion adaptor was moving vertically, instead of having the avatar face the participants and using leftward or rightward motion as adaptors). All of these features ensure that the aftereffects (on discrimination of horizontal test stimuli, following processing of vertical motion adaptors) result from VPT, not otherwise (e.g., not from the self-perspective). Moreover, given that mental-body transformation alone (without motion processing) cannot impact the subsequent motion direction discrimination, the aftereffects observed revealed the effects of processing itself during the mental-representation phase following the mental-body transformation.

In conclusion, we conducted the first research to explore mental representation in level 2 VPT, and showed that perceiving from another's perspective is not the same for everyone. In general people tended to use strategies different from those used in their own perspective, and they then showed facilitated motion processing afterwards. People with increased PT tendencies were more likely to have similar mental representations under the self- and other-perspectives, and exhibited an adaptation aftereffect instead of a facilitation effect on the subsequent motion processing following the VPT task. Future research should continue to examine the mechanisms of aftereffects from viewing motion stimuli under others' perspective among people with high and low PT tendencies and mental representation of level 2 VPT with different tasks.

## Ethics statement

This study was carried out in accordance with the recommendations of the Ethics Review Committee of Peking University with written informed consent from all subjects. All subjects gave written informed consent in accordance with the Declaration of Helsinki. The protocol was approved by the Ethics Review Committee of Peking University.

## Author contributions

XY and NW are equal contributors to this paper (co-first authors). Conception and design of the study: XY, HG; Programming and data collection: XY; Data analysis and drafting the article: NW, XY, SZ; Data interpretation and construction of the argument: HG, NW, SZ, XY; Critical revision of the article: NW, SZ, HG; Project administration, funding acquisition and supervision of the work: HG. All authors approved the final version of the article for submission.

### Conflict of interest statement

The authors declare that the research was conducted in the absence of any commercial or financial relationships that could be construed as a potential conflict of interest.

## Appendix

### Logistic regression fits to discrimination responses

Logistic regression analysis was conducted on the pooled data from all 25 blocks. The probability of participants' “rightward”^1^ responses ***P*** (***x***) was fitted as a function of the motion coherence of the test stimuli (Equation 1), and plotted for different experimental conditions (Figure 2).

(1) $$P\left(x\right)=\frac{1}{1+e^{-(\alpha +\beta \times x+\gamma_{1}\times A_{1}+\gamma_{2}\times A_{2})}}$$

In the above equation, ***x*** is the motion coherence (positive and negative values indicate right or leftward motion, respectively); For the PT condition, ***A***_**1**_ = +**1** for the upward adaptor, −**1** for the downward adaptor, ***A***_**2**_ = **0**; For the AO condition, ***A***_**2**_ = +**1** for the upward adaptor, −**1** for the downward adaptor, ***A***_**1**_ = **0**; for the baseline, ***A***_**1**_, ***A***_**2**_ = **0**. **α** refers to the overall bias of reporting a particular direction; **β** represents the slope of the function; and **γ** reflects the effect of the experimental manipulation. The value −**2γ_i_/β** indicates the separation of the two curves with different adaptors (−**2γ**_**1**_/**β** for the PT condition, and −**2γ**_**2**_/**β** for the AO condition). If the value is positive, it indicates an adaptation effect, for instance, a smaller probability of “rightward” response after viewing a downward, rather than an upward adaptor; whereas a negative value indicates a facilitation effect, for instance, a larger probability of “rightward” response after viewing a downward, rather than an upward adaptor. This value also indicates how much additional motion coherence is needed for the test stimuli to be perceived as equivalent after the participant viewed an adaptor moving in one direction, compared to the opposite direction. It is indexed as the difference of the motion coherences that yielded 50% “rightward” responses between the paired curves (i.e., between adaptors with opposite moving directions, Figure 2).
